# Population Transformer: Learning Population-level Representations of Intracranial Activity

**Published:** 2024-06-05

**Authors:** Geeling Chau, Christopher Wang, Sabera Talukder, Vighnesh Subramaniam, Saraswati Soedarmadji, Yisong Yue, Boris Katz, Andrei Barbu

**Affiliations:** 1 California Institute of Technology; 2 MIT CSAIL, CBMM

## Abstract

We present a self-supervised framework that learns population-level codes for intracranial neural recordings at scale, unlocking the benefits of representation learning for a key neuroscience recording modality. The Population Transformer (PopT) lowers the amount of data required for decoding experiments, while increasing accuracy, even on never-before-seen subjects and tasks. We address two key challenges in developing PopT: sparse electrode distribution and varying electrode location across patients. PopT stacks on top of pretrained representations and enhances downstream tasks by enabling learned aggregation of multiple spatially-sparse data channels. Beyond decoding, we interpret the pretrained PopT and fine-tuned models to show how it can be used to provide neuroscience insights learned from massive amounts of data. We release a pretrained PopT to enable off-the-shelf improvements in multi-channel intracranial data decoding and interpretability, and code is available at https://github.com/czlwang/PopulationTransformer.

## 1 Introduction

Building effective representations of neural recordings is an important tool in enabling neuroscience research. We are particularly interested in modeling intracranial recordings, which rely on probes placed within the brain to provide high temporal resolution recordings of local neural activity [1, 2]. Because of its dispersed placement within the brain volume, intracranial recordings suffer from data sparsity. Moreover, there is often significant variability in probe placement across subjects [1, 2], leading to high variability in input channel meaning. Historically, constructing decoders from intracranial data has relied on supervised learning [3, 2, 4–6], but this requires experimenters to collect annotated data, which is scarce due to patient availability and labor-intensive labeling.

To improve decoding data-efficiency, self-supervised pretraining on unannotated data can be employed to first learn generic representations of the recordings. This means that the model does not have to use valuable annotated samples to learn how to do feature extraction before it can do classification, improving the reach of neuroscientific research.

In this paper, we are interested in developing generic representations of multi-channel intracranial recordings that enable efficient adaptation to a wide range of downstream decoding tasks. Prior work has shown how to pretrain subject-specific [7] or channel-specific [8] models of intracranial data, but such techniques ignore inter-channel relationships or commonalities that might exist across subjects.

The most general approach would be to pretrain using data from multiple datasets, but would require tackling the aforementioned challenges of sparse electrode coverage and variable electrode placement between subjects.

**Figure 1:**
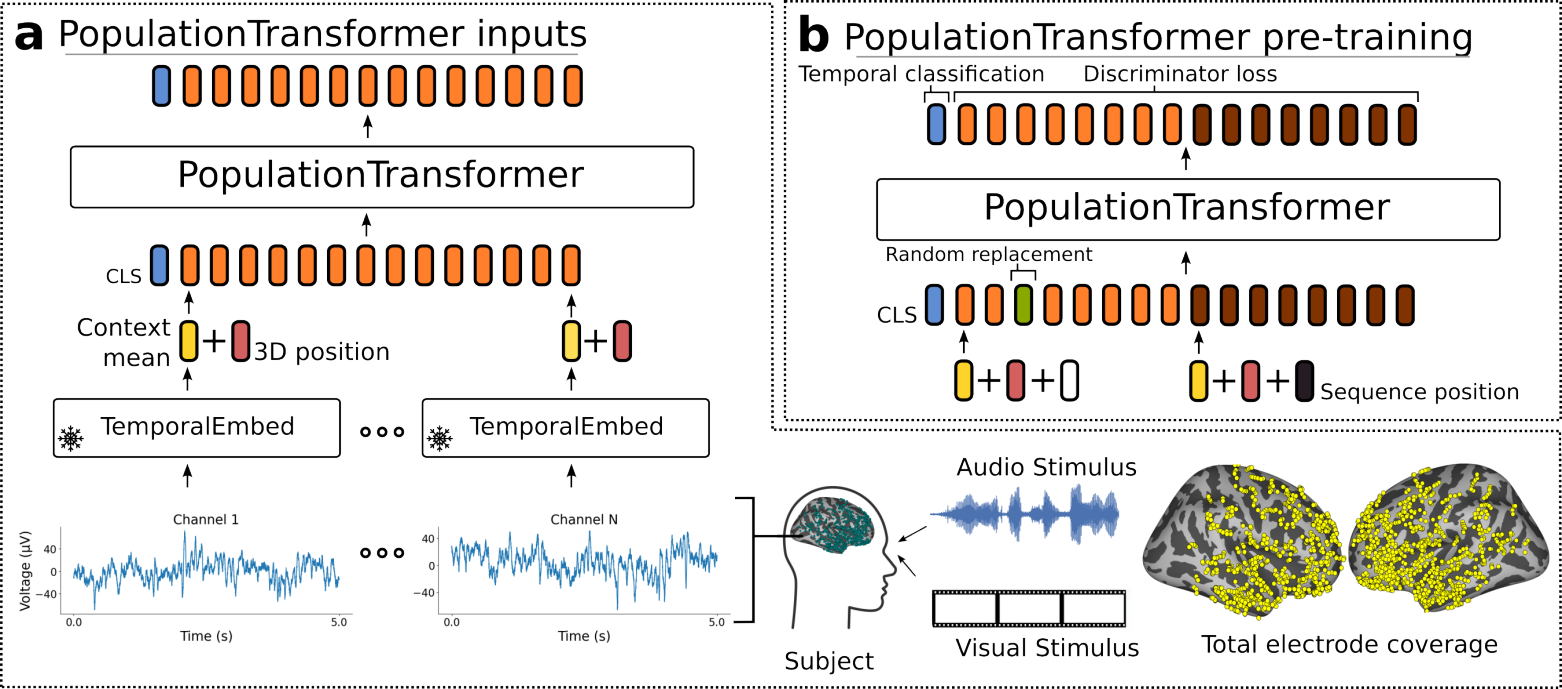
Schematic of our approach. The inputs to our model (a) are the combined neural activities from a collection of intracranial electrodes in a given time interval. These are passed to a frozen temporal embedding model, which produces a set of time-contextual embedding vectors (yellow). The 3D position of each electrode (red) is added to these vectors to produce the model inputs (orange). The PopT produces space-contextual embeddings for each electrode and a [CLS] token (blue), which can be fine-tuned for downstream tasks. During pretraining, (b) the PopT is trained on two objectives simultaneously. In the first, the PopT determines whether two different sets of electrodes (orange vs brown) represent consecutive or non-consecutive times. In the second objective, the PopT must determine whether an input channel has been replaced with activity at a random other time that is inconsistent with the majority of inputs.

We propose Population Transformer (PopT), a self-supervised pretraining approach on transformers [9] that learns subject-generic representations of arbitrary electrode ensembles (Figure 1). During pretraining, we simultaneously optimize both a channel-level and ensemble-level objective, that requires the model to (1) build representations of channels in the context of other channels and (2) meaningfully distinguish temporal relationships between different ensembles of channels.

Our approach builds on top of existing single-channel embeddings, such as BrainBERT [8], which has two key advantages. First, by separating the single-channel embedding and multi-channel-aggregation into different modules, we make our approach agnostic to the specific type of temporal embedding used, leaving room for future independent improvements along either the temporal or spatial dimension, an approach that has been validated in video modeling [10]. Second, by taking advantage of the temporal feature-extraction learned during single-channel pretraining, we make our population-level training more data-efficient.

Empirically, we find that our pretrained PopT outperforms non-pretrained aggregation approaches, highlighting the usefulness of learning spatial relationships during pretraining. Moreover, we find that these benefits hold even for subjects not seen during pretraining, lending to its usefulenss for new subject decoding. We also find that the pretrained PopT weights themselves reveal interpretable patterns for neuroscientific study. We show how the pretrained weights can be probed for connectivity and how the fine-tuned attention weights can be used to map task-specific functional salience.

Our main contributions are:

1. a generic self-supervised learning framework, Population Transformer, PopT, that learns joint representations of varying spatially-sparse time series on top of pretrained single-time-series representations,
2. a demonstration that self-supervised pretraining systematically yields better performance, sample efficiency, and compute efficiency when downstream decoding aggregations of electrode embeddings, even for subjects held out during pretraining,
3. a new method for brain region connectivity analysis and functional brain region identification based on the pretrained and fine-tuned PopT weights,
4. a trained usable off-the-shelf model that computes population-level representations of intracranial neural recordings.

## 2 Related Work

### Self-supervised learning on neural data

Channel independent pretrained models are a popular approach for neural spiking data [11], intracranial brain data [8, 12], and general time-series [13]. Additionally, in fixed-channel neural datasets, approaches exist for EEG [14–16], fMRI [17–19], and calcium imaging [20] datasets. However, all of this work do not learn population-level interactions across datasets with different recording layouts due to the single-channel focus or the ability to assume fixed-channel setups. Several works pretrain spatial and temporal dimensions across datasets with variable inputs [21–25], but most simultaneously learn the temporal embeddings with the spatial modeling, which make them challenging to interpret and computationally expensive to train. As far as we know, we are the first to study the problem of building pretrained channel aggregation models on top of pre-existing temporal embeddings trained across datasets with variable sampling of input channels, allowing for modeling of high quality (>2kHz sampling rate) intracranial data.

### Modeling across variable input channels

Modeling spatial representations on top of temporal embeddings have been found to be beneficial for decoding [3, 7, 26], but prior works use supervised labels, so do not leverage large amounts of unannotated data. The brain-computer-interface field has been studying how to align latent spaces [27–31] which either still requires creating an alignment matrix to learn across datasets or only provides post-training alignment mechanisms rather than learning across datasets. Other approaches impute missing channels or learn latent spaces robust to missing channels [32–34], but these are more suited for the occasional missing channel rather than largely varying sensor layouts. We directly learn spatial-level representations using self-supervised learning across datasets to leverage massive amounts of unannotated intracranial data.

## 3 Population Transformer Approach

In order to learn a subject-generic model of intracranial activity that can handle arbitrary configurations of electrodes, we design a self-supervised training scheme that requires the model to learn representations of individual electrodes as well as groups of electrodes. One component of our self-supervised loss requires the model to identify which channels have been swapped with activity from the same channel, but at a different time point. To do this task, the model must build a representation of the channel’s activity that is sensitive to the context of all the surrounding channels. The other component requires the model to discriminate between randomly selected subsets of electrodes to determine if their activity has occurred consecutively in time or not. This requires the same sensitivity to context, but at the ensemble level. One can think of this swap and discriminate objective as exposing the model to many in-silico ablations of the brain, and asking the model to learn the connections between regions in the presence of these ablations.

A key aspect of our method is the fact that our objective is discriminative, rather than reconstructive, as is often the case in self-supervision [35, 8]. We found this to be necessary, because in practice, the temporal embeddings often have low effective dimension (see [8]), and reconstruction rewards the model for overfitting to “filler” dimensions in the feature vector (see Section 5).

We take additional steps to make our model subject and configuration generic. We provide the absolute position of every electrode to the model, which allows the model to learn a common position embedding space across subjects. We also vary the size of the subsets we select in our sampling procedure to ensure that the model can handle ensembles of differing number, which is important for neuroscience applications, in which experiments have varying number of electrodes, and analysis may be done on the electrode, wire, region, or brain level. Finally, we select that subsets are disjoint, to ensure that the model does not learn to solve the task by trivial copying.

### Architecture

A schematic of our Population Transformer (PopT) approach is shown in Figure 1. Consider a given subject with $N_{c}$ channels indexed by $C=\left\{1,\ldots ,N_{c}\right\}$. Activity from channel i at time t can be denoted by $x_{i}^{t}$. The PopT takes as input an interval of brain activity $X=\left\{x_{i}^{t}|i\in C\right\}$ from a given time t and a special [CLS] token. Per channel, each interval of brain activity is passed through a temporal embedding model T, in our case BrainBERT, to obtain a representation of each channel’s temporal context.

Before being inputted to the PopT, each channel’s 3D position is added to this embedding, so the final input is $X_{B}=\{T(x)+pos(i)+𝒩(0,\sigma )|x\in X\}$. Here, we add Gaussian fuzzing to prevent overfitting to a particular set of coordinates. Spatial location is given by the electrode’s Left, Posterior, and Inferior coordinates [36]; see [8] for details on how these were obtained. Each coordinate is encoded using sinusoidal position encoding [9]. And the three encodings are concatenated together to form the position embedding $pos(i)=\left[e_{\text{left}};e_{\text{post}.};e_{\text{inf}}\right]$.

The core of PopT consists of a transformer encoder stack (see Appendix A: Architectures). The output of the PopT are spatial-contextual embeddings of the channels $Y=\left\{y_{i}\right\}$ as well as an embedding of the CLS token $y_{cls}$. During pretraining, the PopulationTransformer additionally is equipped with a linear layer head for the [CLS] token output and separate linear layer heads for all other individual token outputs. These produce the scalars ${\tilde{y}}_{cls}$ and ${\tilde{y}}_{i}$ and respectively, which are used in the objective (see Figure 1a).

### Pretraining

Our pretraining objective has two components: channel-wise discrimination and next brain state discrimination, which is a group-level objective (see Figure 1b). First, we describe the next brain state discrimination task. Two different subsets of channels $S_{A},S_{B}\subset C$ are chosen with the condition that they be disjoint $S_{A}\cap S_{B}=\emptyset$. During pretraining, the model receives the activities from these channels at separate times $X_{A}=\left\{x_{i}^{t}|i\in S_{A}\right\}$ and $X_{B}=\left\{x_{i}^{t^{\prime }}|i\in S_{B}\right\}$. The objective of the task is then to determine whether these states $X_{A}$ and $X_{B}$ have occurred consecutively in time or are separated by some further, randomly selected interval. Given the output of the classification head, the objective is the binary cross entropy: $\frac{1}{N_{\text{batch}}}\sum_{i}\;y_{i}^{*}{ℒ}_{N}=y_{cls}^{*}log\left(p\left({\tilde{y}}_{cls}\right)\right)+\left(1-y_{cls}^{*}\right)log\left(p\left({\tilde{y}}_{cls}\right)\right)$ where $y_{cls}^{*}=1\left(\left|t-t^{\prime }\right|<500ms\right)$.

Next we describe our channel-wise discriminative learning. The token level objective is to determine whether a channels activity has been swapped with activity from a random time. Precisely, activity from each channel i is drawn from a time $t_{i}$. All channels are drawn from the same time $t_{i}=T$, and then 10% of the channels are randomly selected to have their activity replaced with activity from the same channel, but taken from a random point in time $t_{i}\neq T$. Then, given the token outputs of the Population Transformer, the objective function is the binary cross entropy: ${ℒ}_{C}=\frac{1}{N_{\text{batch}}}\sum_{i}\;y_{i}^{*}log(p(\tilde{y}))+\left(1-y_{i}^{*}\right)\;log\left(p\left({\tilde{y}}_{i}\right)\right)$ where $y_{i}^{*}=1\left(t_{i}\neq t\right)$.

Then, our complete objective function is $ℒ={ℒ}_{N}+{ℒ}_{C}$.

### Fine-tuning

During fine-tuning, the [CLS] intermediate representation, ${\tilde{y}}_{cls}$ of the pretrained PopT is passed through a single layer linear neural network to produce a scalar ${\overset{ˆ}{y}}_{cls}$. This scalar is the input to binary cross entropy loss for our decoding tasks (see Section 4).

## 4 Experiment Setup

### Data

We use the publicly available subject data from [8]. Data was collected from 10 subjects (total 1,688 electrodes, with a mean of 167 electrodes per subject) who watched 26 movies while intracranial probes recorded their brain activity. The movie transcripts were aligned to the brain activity so that features such as volume, pitch, etc. could be associated with the corresponding sEEG readings. 19 of the sessions are used for pretraining. 7 of the sessions are held-out for evaluation.

### Decoding

We evaluate the effectiveness of our pretrained PopT model by fine-tuning it on the four downstream decoding task used in the evaluation of [8]. Two of the tasks are audio focused: determining whether a word is spoken with a high or low pitch and determining whether a word is spoken loudly or softly. And two of the tasks have a more linguistic focus: determining whether the beginning of a sentence is occurring or determining whether any speech at all is occurring.

Our approach enables decoding on any arbitrary size of ensemble. We verify that our model is able to leverage additional channels for improved decoding performance that scales the number of inputs. To test this, we first order the electrodes by their individual linear decodability per task, and we increase the number of channels available to the model at fine-tuning time.

### Baselines

We want to determine whether the information about spatial relationships learned during pretraining was useful at fine-tuning time. For comparison, we concatenate the raw BrainBERT embeddings and train a linear and deep NN on the decoding task. This sets a baseline for how much improvement is achievable from simply having more channels available at once. To determine whether our performance can be attributed to using a more powerful architecture, we also fine-tune a PopT without pretraining, i.e. with randomly initialized weights.

## 5 Results

### Decoding performance

**Figure 2:**
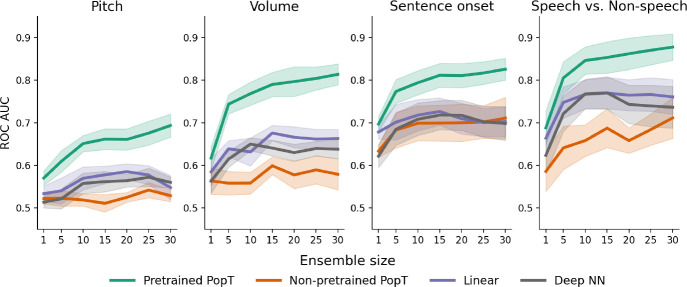
Pretrained PopT downstream performance scales better with ensemble size. Increasing channel ensemble size from 1 to 30 (x-axis), we see pretrained PopT (green) decoding performance (y-axis) not only beat non-pretrained approaches (orange, purple, pink), but also continually improve more with increasing channel count. Shaded bands show the standard error across subjects.

Compared to trying to decode from the bare BrainBERT embeddings or from a non-pretrained PopT, the PopT both achieves better decoding performance (see Table 1) and does so with steeper scaling per added channel (Figure 2).

To verify that the weights of the pretrained PopT capture neural processing well even without fine-tuning, we also train a linear-encoder on top of the frozen PopT [CLS] token and find the same trends ( Figure 10: Frozen scaling – Figure 10). This point in particular is important in building confidence in the results of our interpretability studies (see Section 6), in which we use the frozen pretrained weights to analyze connectivity.

### Sample and compute efficiency

**Figure 3:**
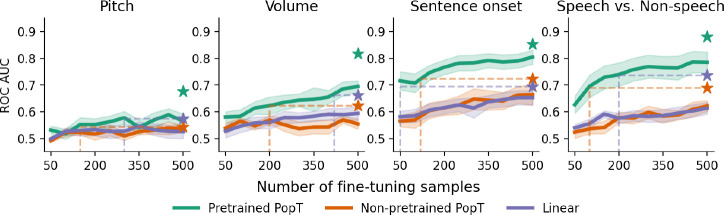
Pretrained PopT is more sample efficient when fine-tuning. Varying the number of samples available to each model at train time (x-axis), we see how the pretrained PopT is highly sample efficient, requiring only a fraction of samples to reach the full performance level of non pretrained aggregation approaches (dashed lines). Bands show standard error across test subjects. Stars indicate performance of the model trained on the full fine-tuning dataset.

**Figure 4:**
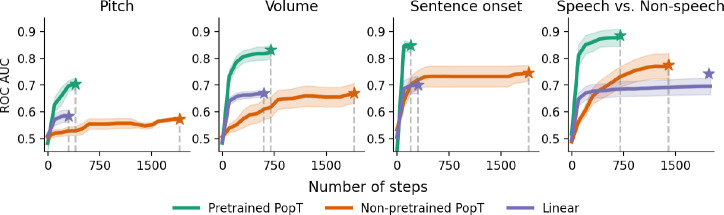
Pretrained PopT is consistently more compute efficient when fine-tuning. Number of steps required for each model to reach final performance during fine-tuning (dashed lines). We find that pretrained PopT consistently requires fewer than 750 steps (each step is training on a batch size of 256) to converge, in contrast to the 2k steps required for the non pretrained PopT. Linear aggregation can be similarily compute efficient, but occasionally benefits from more training steps depending on dataset size (Speech vs. Non-speech). Bands show standard error across test subjects. Stars indicate fully trained performance.

Our PopT learns spatial relationships between channels, in a way that makes downstream supervised learning more data and compute efficient (see Figure 3 and Figure 4). Compared to the non-pretrained baseline models, fine-tuning the pretrained PopT achieves more decoding performance from fewer samples. At only 200 examples, the pretrained PopT has already surpassed the performance achieved by the non-pretrained model on the full dataset, for the volume, sentence onset, and speech vs. non-speech tasks Figure 3. The number of steps required for each model to converge is also greatly reduced by starting with the pretrained PopT. We see that fine-tuning the pretrained PopT consistently requires 500 steps or fewer steps to reach its converged performance Figure 4, whereas the non pretrained baselines may require 2k or more steps.

### Generalizability

**Figure 5:**
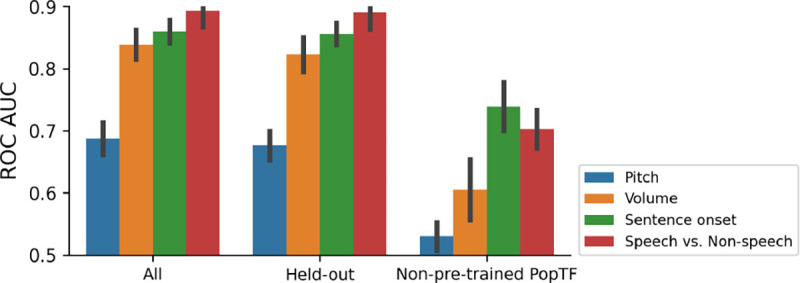
Gains in decoding performance are available to new subjects. To test whether our pretrained PopT weights will be able to yield decoding benefits for unseen subjects, we run a hold-one-out analysis in which we exclude one subject from pretraining and then evaluate on that subject during fine-tuning (Held-out). We compare this with our full PopT model that has seen all subjects during pretraining (All). A minimal decrease in downstream decoding performance is found if the subject is held-out from pretraining (Held-out vs All). This is in stark contrast to the achievable downstream performance with a non pretrained PopT (Non-pretrained PopT).

To test whether our pretrained weights will be useful for subjects not seen during training, we conduct a hold-one-out analysis. We pretrain a model using all subjects except for one, and then fine-tune and evaluate on the model downstream. We find that missing a subject from pretraining does not significantly affect the downstream results (see Figure 5). This raises our confidence that the pretrained weights will be useful for unseen subjects and for researchers using new data.

### Scaling with number of pretraining subjects

**Figure 6:**
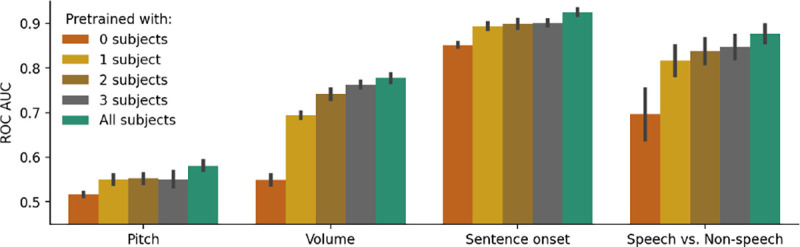
Pretraining with more subjects leads to better downstream performance. We pretrain PopT with different number of subjects (colors) and test on our decoding tasks (x-axis). Bars indicate mean and standard error of performance across channel ensembles 5–30 on test subject 3. Model descriptions: 0 subjects (non-pretrained), 1 subject (pretrain w/ subject 4), 2 subjects (pretrain w/ subjects 4, 8), 3 subjects (pretrain w/ subjects 4, 8, 10), All subjects (pretrain w/ all 10 subjects). Pretraining with one subject gives a considerable benefit compared to no pretraining (red to yellow), but the addition of more subjects to pretraining consistently improves performance (yellow → green).

To investigate the effect of scaling pretraining data on our model, we pretrain additional versions of PopT using only 1, 2, or 3 subjects. We find a consistent improvement in downstream decoding when we increase the number of pretraining subjects available across all our downstream decoding tasks Figure 6. A significant improvement is found with just 1 pretraining subject already, potentially due to adaption to the temporal embeddings used. The decoding performance using all our pretraining data is significantly higher in most decoding tasks than with just 1 or 2 subjects in the pretraining data, suggesting the potential for our framework to continue scaling with more subjects.

### Ablation of loss components and position information

An ablation study confirms that both the network-wise and channel-wise component of the pretraining objective contribute to the downstream performance (Table 2). We also find that including the 3D position information for each channel is critical for decoding. These findings also hold when the PopT is kept frozen during fine-tuning (see Appendix G: Frozen ablation – Table 4). Additionally, we find that the discriminative nature of our loss is necessary for decoding. Attempting to add an L1 reconstruction term to our pretraining objective results in poorer performance, perhaps because the model learns to overfit on low-entropy features in the embedding. Our discriminative loss requires the model to understand the embeddings in terms of how they can be distinguished from one another, which leads the model to extract more informative representations.

## 6 Interpreting Learned Weights

Our final analysis are two interpretability studies of the Population Transformer’s learned weights. In the first, we use the PopT weights to uncover connectivity maps of the channels, and in the second, we use the attention weights of the fine-tuned PopT to identify candidate functional brain regions per decoding task.

### Connectivity

**Figure 7:**
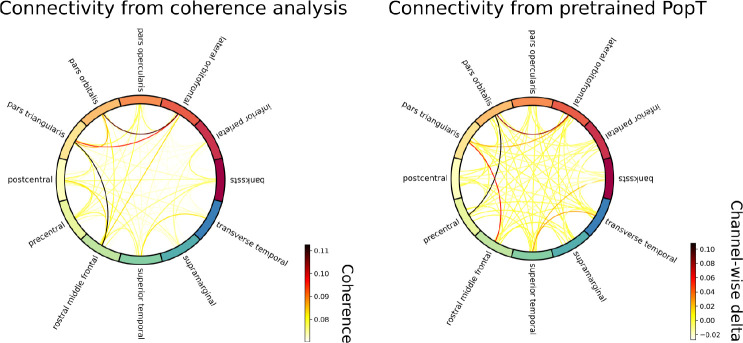
Probing the pretrained model for inter-channel connectivity Traditionally, connectivity analysis between regions is done by computing the coherence [37], i.e. cross-correlation, between electrode activity (left). We propose an alternative analysis based on how channels matter to each other in the context of our pretraining objective. Iteratively, we select an electrode, mask out its activity, and then plot the degradation in the channel-wise objective function of the pretrained PopT objective for the unmasked electrodes. Plotting the values of this delta (right) recovers the main points of connectivity, purely based off of the relationships learned during pretraining. Shown here is a plot for a single subject; plots for all test subjects can be seen in Appendix E: Connectivity.

For identifying connectivity per region, traditional neuroscience analyses typically use cross-correlation as a measure of channel connectivity [37]. Our PopT allows for an alternative method of determining connectivity, based on the degree to which channels are sensitive to each other’s context. We go through our channels, masking one channel and then evaluating the model’s performance on the pretraining channel-wise objective for the remaining unmasked channels. We take the degradation in performance as a measure of connectivity. We can construct plots as in Figure 7, that recapitulate the strongest connectivity of the cross-correlation maps. Note that while some approaches for modelling brain activity explicitly build this into their architecture [25] we recover these connections purely as a result of our self-supervised learning.

### Candidate functional brain regions from attention weights

**Figure 8:**
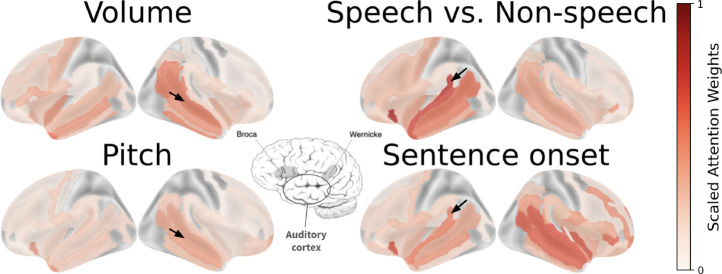
Attention weights from a fine-tuned PopT identify candidate functional brain regions Candidate functional maps can be read from attention weights of a PopT fine-tuned on our decoding tasks. For the Volume and Pitch tasks, note the weight placed on the primary auditory cortex (black arrows), but not in Wernicke’s area. For the Speech vs Non-speech and Sentence onset tasks, note the weight placed on regions near Wernicke’s area (black arrows). Center brain figure highlight regions related to auditory-linguistic processing such as language production area Broca’s area, language understanding Wernicke’s area, and the primary auditory cortex (adapted from [40]).

**Figure 10:**
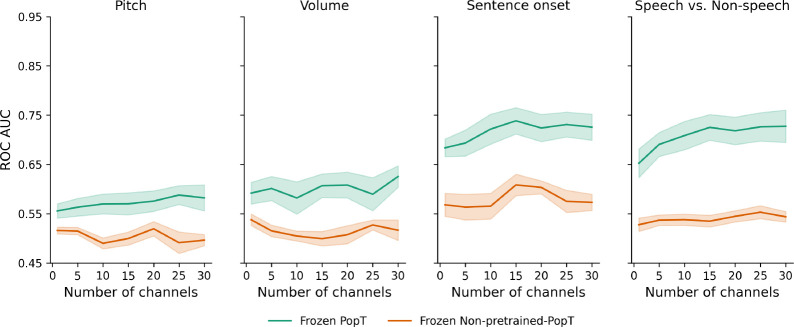
Pretraining is critical to frozen PopT performance that scales with the number of channels. As in Figure 2, we see that pretraining results in better downstream decoding and better scaling with the number of added channels. However, unlike in Figure 2, the PopT weights are frozen during fine-tuning, and only the linear classification head is updated. Bands show standard error across subjects. Results are shown for a frozen PopT with BrainBERT inputs.

Next, we discuss the possibility of uncovering functional brain regions from the attention weights. After fine-tuning our weights on a decoding task, we can examine the attention weights of the [CLS] output for candidate functional brain regions. We obtain a normalized Scaled Attention Weight metric across all subjects to be able to analyze candidate functional brain regions across sparsely sampled subject datasets Figure 8. The Scaled Attention Weight is computed from raw attention weights at the [CLS] token passed through the attention rollout algorithm [38]. The resulting weights from each channel are then grouped by brain region according to the Destrieux layout [39]. Additional details available in Appendix D.

The resulting weights reveal expected functional brain regions related to the tasks decoded Figure 8. For our low-level perceptual auditory tasks (Volume and Pitch), we see that our model learns to attend to the primary auditory cortex. For our higher-level language distinction tasks (Speech vs. Non-speech and Sentence onset), we see higher attention is placed at language areas like Wernicke’s area. Given the massive pretraining PopT undergoes, these scaled attention weights provide a valuable a new tool for discovering candidate functional brain regions.

## 7 Discussion

We presented a self-supervised learning scheme for learning effective representations of intracranial activity from temporal embeddings. We find that pretraining the PopT results in better channel efficiency at fine-tuning time. This can reduce the number of electrodes needed in future experiments, which is critical for an invasive recording modality such as sEEG. We showed that self-supervised pretraining imbues our model with knowledge of spatial relationships between these embeddings and improved downstream decoding that scales with the number of available channels. As an aside, we note that the tasks we evaluate necessitate wide coverage of the brain. This is evidenced by the fact that performance scales with the number of input channels. With future collection of high quality intracranial data, we can continue scaling PopT and uncover exciting new data-driven findings for neuroscience.

By decoupling temporal and spatial feature extraction, we are able to leverage existing temporal embeddings to learn spatiotemporal representations efficiently and with a smaller number of parameters. Our approach also leaves open the possibility for independent improvement in temporal modeling. If future approaches introduce better time-series representations, are approach will be able to incorporate these advantages directly. Finally, we note that our method can serve more generally as a representation learning approach for any ensemble of sparsely distributed time-series data channels.

### Limitations and Future Work

As far as we know, no large public sEEG dataset that are of the same level of quality as ours (2048 Hz sampling rate, aligned electrode coordiantes, multimodal stimulus) are available, so direct comparison with existing approaches is difficult. Additionally, existing sEEG test datasets that have been used by existing deep learning models [21] focus on the artifact and seizure detection tasks [41], which are less interesting at a network-level due to the dependence on human labeling while looking at the time-series sEEG data [42].

Given the high sampling rate of our sEEG data (10x of prior work [21, 25]), training an end-to-end spatio-temporal model on our data would not have been computationally feasible, lending to the benefits of learning spatial representations on top of learned temporal embeddings. With the development and acquisition of compute resources, it would be a valuable future work to compare our approach with end-to-end approaches.

## 8 Conclusion

We introduced a pretraining method for learning representations of arbitrary ensembles of intracranial electrodes. We showed that our pretraining produced considerable improvements in downstream decoding, that would not have been possible without the knowledge of spatial relationships learned during the self-supervised pretraining stage. We showed that this scheme produces interpretable weights from which connectivity maps and candidate functional brain regions can be read. Finally, we release the pretrained weights for our PopT with BrainBERT inputs as well as our code for plug-and-play pretraining with any temporal embedding (see attached supplemental materials).

## Figures and Tables

**Table 1:** Pretraining PopT is critical to downstream decoding performance. We test on a variety of audio-linguistic decoding tasks (see Section 4) with either a single channel (row 1) or 90 channels (rows 2–5) as input. Shown are the ROC-AUC mean and standard error across subjects. We see that all aggregation approaches (rows 2–5) outperform single-channel decoding with BrainBERT [8] (row 1). Pretraining PopT and then fine-tuning it for downstream decoding results in significantly better performance (bold) compared to non-pretrained aggregation approaches (rows 2–4). This gain cannot be explained by simply providing more temporal embeddings, as evidenced by the performance of Linear and Deep NN (rows 2 and 3) that take the concatenated raw temporal embeddings as input. Neither can the gain be attributed to simply using a Transformer architecture, as is shown by a comparison with a non-pretrained PopT (row 4).

	Pitch	Volume	Sent. Onset	Speech/Non-speech

BrainBERT: single channel	0.53 ± 0.05	0.58 ± 0.08	0.68 ± 0.04	0.66 ± 0.09
Linear + BrainBERT	0.59 ± 0.08	0.66 ± 0.08	0.70 ± 0.09	0.71 ± 0.11
Deep NN + BrainBERT	0.58 ± 0.08	0.67 ± 0.08	0.71 ± 0.10	0.72 ± 0.10
Non-pretrained PopT	0.53 ± 0.06	0.61 ± 0.13	0.74 ± 0.10	0.70 ± 0.08
Pretrained PopT	**0.69 ± 0.07**	**0.84 ± 0.06**	**0.86 ± 0.05**	**0.89 ± 0.07**

**Table 2:** PopT ablation study. We individually ablate our losses and positional encodings during pretraining then decode on the resulting models. Shown are ROC-AUC mean and standard error across subjects. The best performing model across all decoding tasks uses all three of our proposed components, showing that they are all necessary. Removing our positional encoding during pretraining and fine-tuning drops the performance the most, indicating that position encoding is highly important for achieving good decoding. Additionally, we attempt adding a reconstruction component to the loss as a regularizing term, but find that this leads to poorer performance (see section 5).

	Pitch	Volume	Sent. Onset	Speech/Non-speech

PopT	**0.69 ± 0.07**	**0.84 ± 0.06**	**0.86 ± 0.05**	**0.89 ± 0.07**
PopT w/o group-wise loss	0.66 ± 0.07	0.83 ± 0.06	0.84 ± 0.04	0.88 ± 0.08
PopT w/o channel-wise loss	0.67 ± 0.06	0.81 ± 0.08	0.84 ± 0.06	0.87 ± 0.09
PopT w/o position encoding	0.59 ± 0.07	0.67 ± 0.10	0.75 ± 0.08	0.79 ± 0.08
PopT with reconstruction loss	0.60 ± 0.11	0.73 ± 0.11	0.81 ± 0.05	0.83 ± 0.09

**Figure 9:**
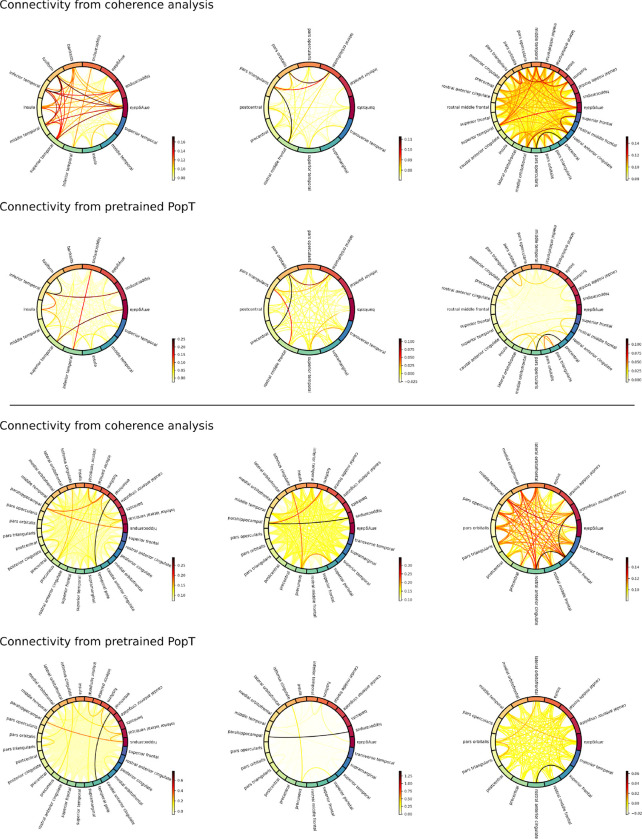
Full connectivity for our 7 test subjects. We compare between traditional connectivity analaysis performed via coherence (top row in each section) and the analysis based on our PopT pretrained weights (bottom row in each section). We note that our analysis usually recovers the strongest points of connectivity fromt the traditional analysis. Coherence was computed using scikit-learn’s [49] signal.coherence.

## A Architectures and training

### Pretrained PopT

The core Population Transformer consists of a transformer encoder stack with 6 layers, 8 heads. All layers (N=6) in the encoder stack are set with the following parameters: $d_{h}=512$, H=8, and $p_{\text{dropout}}=0.1$. We pretrain the PopT model with the LAMB optimizer [43] (lr=1e-4), with a batch size of $n_{\text{batch}}=256$, and train/val/test split of 0.98, 0.01, 0.01 of the data. We pretrain for 500,000 steps, and record the validation performance every 1,000 steps. Downstream evaluation takes place on the weights with the best validation performance. We use the intermediate representation at the [CLS] token $d_{h}=512$ and put a linear layer that outputs to $d_{\text{out}}=1$ for fine-tuning on downstream tasks. These parameters for pretraining were the same for any PopT that needed to be pretrained (hold-one-out subject, subject subsets, ablation studies).

### Non-pretrained PopT

The architecture for the non-pretrained PopT is the same as the pretrained PopT (above). However, no pretraining is done, and the weights are randomly initialized with the default initializations.

### Linear

The linear baseline consists of a single linear layer that outputs to $d_{\text{out}}=1$. The inputs are flattened and concatenated BrainBERT embeddings $d_{emb}=756$ from a subset of channels S⊂C. Thus, the full input dimension is $d_{\text{input}}=d_{\text{emb}}\;*\;|S|$.

### Deep NN

The inputs are the same as above, but the decoding network now consists of 5 stacked linear layers, each with $d_{h}=512$ and a GeLU activation.

### Downstream Training

For both PopT models, we train with these parameters: AdamW optimizer [44], $lr=5e^{-4}$ where transformer weights are scaled down by a factor of 10 $\left(lr_{t}=5e^{-5}\right),\;n_{\text{batch}}=256$, a Ramp Up scheduler [45] with warmup 0.025 and Step LR gamma 0.99, reducing 100 times within the 2000 total steps that we train for. For Linear and DeepNN models, we train with these parameters: AdamW optimizer [44], $lr=5e^{-4}$, $n_{\text{batch}}=256$, a Ramp Up scheduler [45] with warmup 0.025 and Step LR gamma 0.95, reducing 25 times within the 17,000 total steps we train for. For all downstream decoding, we use a fixed train/val/test split of 0.8, 0.1, 0.1 of the data.

### Compute Resources

To run all our experiments (data processing, pretraining, evaluations, interpretability), one only needs 1 NVIDIA Titan RTXs (24GB GPU Ram) and up to 80 CPU cores (700GB memory) if running sequentially. Pretraining PopT takes 4 days on 1 GPU. Our downstream evaluations take a few minutes to run each. For the purposes of data processing and gathering all the results in the paper, we parallelized the experiments on roughly 8 GPUs and 80 CPU cores.

## B Decoding tasks

We follow the same task specification as in Wang et al. [8], with the modification that the pitch and volume examples are determined by percentile (see below) rather than standard deviation in order to obtain balanced classes.

### Pitch

The PopT receives an interval of activity and must determine if it corresponds with a high or low pitch word being spoken. For the duration of a given word, pitch was extracted using Librosa’s piptrack function over a Mel-spectrogram (sampling rate 48,000 Hz, FFT window length of 2048, hop length of 512, and 128 mel filters). For this task, for a given session, positive examples consist of words in the top-quartile of mean pitch and negative examples are the words in the bottom quartiles.

### Volume

The volume of a given word was computed as the average intensity of root-mean-square (RMS) (rms function, frame and hop lengths 2048 and 512 respectively). As before, positive examples are the words in the top-quartile of volume and negative examples are those in the bottom quartiles.

### Speech vs non-speech

Positive examples are intervals of brain activity that correspond with dialogue being spoken in the stimuli movie. Negative examples are intervals of activity from 1s periods during which no speech is occurring in the movie.

### Sentence onset

Negative examples are as before. Positive examples are intervals of brain activity that correspond with hearing the first word of a sentence.

## C Data

**Table 3:** Subject statistics Subjects used in PopT training, and held-out downstream evaluation. Table taken from [8]. The number of uncorrupted, electrodes that can be Laplacian re-referenced are shown in the second column The average amount of recording data per subject is 4.3 (hrs).

Subj.	Age (yrs.)	# Electrodes	Movie	Recording time (hrs)	Held-out

1	19	91	Thor: Ragnarok	1.83	
			Fantastic Mr. Fox	1.75	
			The Martian	0.5	x

2	12	100	Venom	2.42	
			Spider-Man: Homecoming	2.42	
			Guardians of the Galaxy	2.5	
			Guardians of the Galaxy 2	3	
			Avengers: Infinity War	4.33	
			Black Panther	1.75	
			Aquaman	3.42	x

3	18	91	Cars 2	1.92	x
			Lord of the Rings 1	2.67	
			Lord of the Rings 2 (extended edition)	3.92	

4	9	135	Megamind	2.58	
			Toy Story	1.33	
			Coraline	1.83	x

5	11	205	Cars 2	1.75	x
			Megamind	1.77	

6	12	152	Incredibles	1.15	
			Shrek3	1.68	x
			Megamind	2.43	

7	6	109	Fantastic Mr. Fox	1.5	

8	4.5	72	Sesame Street Episode	1.28	

9	16	102	Ant Man	2.28	

10	12	173	Cars 2	1.58	x
			Spider-Man: Far from Home	2.17	

## D Interpretation Methods

### Connectivity analysis

We start with a pretrained PopT. To test a particular channel’s contribution to connectivity, we mask it with all zeros. Then, we consider the remaining unmasked channels and ask, how much increase do we see in the pretraining channel-wise loss? Recall that this objective is to determine whether or not a channel has had its inputs swapped with random activity. If the increase in loss is large, then we infer that the masked channel provided important context for this task. Using this delta as a measure for connectivity, we can then average across regions, as provided by the Desikan-Killiany atlas [46] and produce a plot using mne-connectivity [47].

### Scaled Attention Weight

First, we obtain an attention weight matrix across all trials which includes weights between all tokens. Then, we perform attention rollout [38] across layers to obtain the contributions of each input channel by the last layer. We take the resulting last layer of rollout weights for all channels, where the target is the [CLS] token, normalize within subject, and scale by ROC AUC to obtain the Scaled Attention Weight per channel. Finally, we plot the 0.75 percentile weight per region, as mapped by the Destrieux atlas [39] using Nilearn [48].

## G Frozen ablation

**Table 4:** An ablation study of the components of our approach for the frozen PopT. During pretraining, we alternate using either only the CLS or token contrastive component of the loss. We fine-tune these weights on all subjects. We find that both components contribute to the full model’s performance.

	Sentence onset	Speech/Non-speech	Pitch	Volume

Frozen PopT	**0.73 ± 0.06**	**0.72 ± 0.08**	0.59 ± 0.06	**0.63 ± 0.07**
w/o cls	0.67 ± 0.08	0.68 ± 0.07	0.58 ± 0.04	0.60 ± 0.07
w/o replace loss	0.69 ± 0.07	0.69 ± 0.09	**0.59 ± 0.06**	0.62 ± 0.06
w/o position encoding	0.70 ± 0.07	0.69 ± 0.07	0.56 ± 0.08	0.61 ± 0.06
